# Diversity of benthic marine mollusks of the Strait of Magellan, Chile (Polyplacophora, Gastropoda, Bivalvia): a historical review of natural history

**DOI:** 10.3897/zookeys.963.52234

**Published:** 2020-08-24

**Authors:** Cristian Aldea, Leslie Novoa, Samuel Alcaino, Sebastián Rosenfeld

**Affiliations:** 1 Centro de Investigación GAIA Antártica, Universidad de Magallanes, Av. Bulnes 01855, Punta Arenas, Chile; 2 Departamento de Ciencias y Recursos Naturales, Universidad de Magallanes, Chile; 3 Facultad de Ciencias, Laboratorio de Ecología Molecular, Departamento de Ciencias Ecológicas, Universidad de Chile, Santiago, Chile; 4 Laboratorio de Ecosistemas Marinos Antárticos y Subantárticos, Universidad de Magallanes, Chile; 5 Instituto de Ecología y Biodiversidad, Santiago, Chile

**Keywords:** benthos, Magellanic Biogeographic Province, Mollusca, South Atlantic, South Pacific, species richness

## Abstract

An increase in richness of benthic marine mollusks towards high latitudes has been described on the Pacific coast of Chile in recent decades. This considerable increase in diversity occurs specifically at the beginning of the Magellanic Biogeographic Province. Within this province lies the Strait of Magellan, considered the most important channel because it connects the South Pacific and Atlantic Oceans. These characteristics make it an interesting area for marine research; thus, the Strait of Magellan has historically been the area with the greatest research effort within the province. However, despite efforts there is no comprehensive and updated list of the diversity of mollusks within the Strait of Magellan up to now. This study consisted of a complete bibliographic review of all available literature that included samples of mollusks in the Strait of Magellan. More than 300 articles were reviewed, covering 200 years of scientific knowledge. There were 2579 records belonging to 412 taxa, of which 347 are valid species. Of the total valid species, 44 (~13%) are considered of doubtful presence in the Strait. This work increases the known richness of mollusks of the Strait of Magellan by 228%; it is also the first report that integrates all available diversity studies of the three most speciose classes of benthic mollusks (Gastropoda, Bivalvia and Polyplacophora) from the Strait of Magellan.

## Introduction

It has been described that mollusks show an increase in diversity towards high latitudes in the Chilean southeastern Pacific coast (Valdovinos et al. 2003). This increase in mollusk richness occurs around 42°S, coinciding with the beginning of the Magellanic Biogeographic Province (Spalding et al. 2007). The Magellanic Province has been the focus of study of several scientific expeditions that contributed to the knowledge of marine mollusks. The first reports were made by King and Broderip (1832), d’Orbigny (1835–1846) and Philippi (1845). Other reports that contributed considerably to the knowledge of mollusks of the Magellanic Province were Smith (1881), Rochebrune and Mabille (1889), Strebel (1904, 1905a, b, 1906, 1907, 1908), Odhner (1926), Marcus (1959) and Soot-Ryen (1959). Carcelles and Williamson (1951) published the first checklist of species of marine mollusks of the Magellanic Province in the 1950s, defining the province from around 37°S in the Pacific coast and 43°S in the Atlantic coast, to 56°S. In their checklist 614 species were reported. Many taxonomic revisions of specific groups have been published (e.g., McLean 1984a; Castellanos 1988; Castellanos and Landoni 1988, 1989, 1990, Castellanos 1990, 1992a, b; Castellanos and Landoni 1993a, b; Castellanos et al. 1993; Ponder and Worsfold 1994; Schrödl 1996), therefore the checklist of Carcelles and Williamson (1951) had to be updated, for species synonyms and newly found species. Linse (1999) presented a new checklist of mollusks of the Magellanic Province, defining the province from around 41°S in the Pacific and Atlantic coasts to 56°S. However, the classes Polyplacophora and Cephalopoda were excluded from this checklist, which included 397 species of mollusks.

One of the most important channels in the Magellanic Province is the Strait of Magellan, where most historical reports of mollusks are focused. This extensive channel connects the Pacific and Atlantic Oceans and is considered the most important one of the province. It is influenced by water masses of the Pacific, Atlantic and Southern Oceans, and it possess several geological characteristics derived from the last glaciation (Antezana 1999). For these reasons the Strait of Magellan offers unique characteristics for the study of biodiversity and related aspects of the biogeography of mollusks (Linse et al. 2006). Linse et al. (2006) presented the only report of mollusk richness in the Strait of Magellan, which contains 116 species. However, a list of species is not provided and only the classes Gastropoda and Bivalvia are included. Between the year 2000 and the present there have been several studies that have provided more information about the diversity of mollusks in the Strait of Magellan (e.g., Ríos et al. 2003; Ríos et al. 2005; Ríos et al. 2007; Thatje and Brown 2009; Aldea et al. 2011; Rosenfeld et al. 2013; Rosenfeld et al. 2015), presenting new records of species. Several taxonomic revisions of specific groups have been published in recent years, where erroneous records, changes in nomenclature, synonymized species and descriptions of new species have been made (e.g., Sirenko 2006a; Zelaya and Geiger 2007; Aranzamendi et al. 2009; Zelaya 2009; González-Wevar et al. 2011; Güller et al. 2016; Pastorino 2016; Güller and Zelaya 2017; Korshunova et al. 2017). In order to have a comprehensive list of species in the most important channel of the Magellanic Province it is necessary to provide an updated list of records of the malacofauna of the Strait of Magellan. The objective of this study is to provide the first list of species of benthic marine mollusks of the three most speciose and best documented classes (Polyplacophora, Gastropoda, Bivalvia) of the Strait of Magellan, integrating all studies throughout history.

## Materials and methods

**Figure 1.**
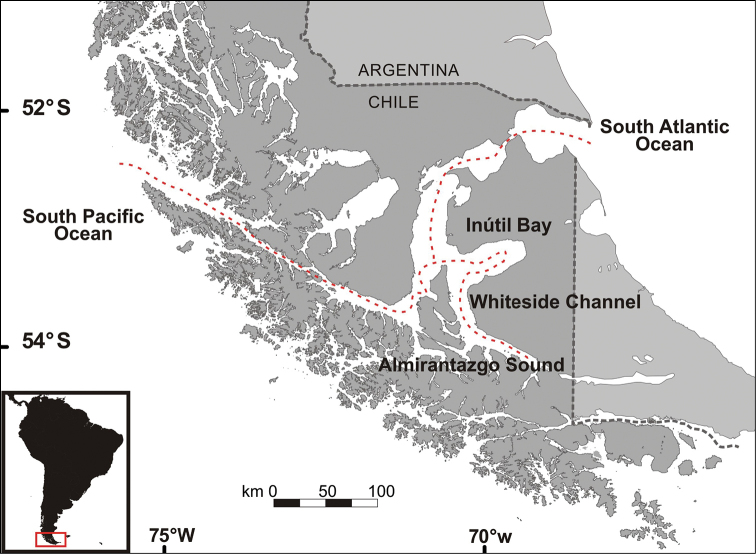
Study area. Location of the Strait of Magellan (marked by the red line), including Inútil Bay, Whiteside Channel and Almirantazgo Sound.

To make the list of mollusks as complete as possible, information was gathered from all the available scientific publications that have sampled or reviewed benthic marine mollusks in the Magellanic Province, from the expedition of the HMS Beagle in the 19^th^ century (King and Broderip 1832) to the present. A total of 323 articles were reviewed, of which 146 contained species within the Magellanic Province. The records and their respective geographical positions were entered into a spreadsheet structured with the Darwin Core Standard (Wieczorek et al. 2012), adjusted taxonomically according to the MolluscaBase (2019) and the revisions of classification and systematics of gastropods (Bouchet et al. 2017), bivalves (Nevesskaja 2009) and polyplacophorans (Sirenko 2006b). The Strait of Magellan was divided into 420 quadrants of 6×6 minutes of latitude and longitude. The records located within this area were analyzed (Fig. 1), taking into account their georeference or approximate location. This analysis was developed using tools for Google Earth (http://www.earthpoint.us), which transforms XLS extension files (Excel format) to KML (files that contains geographic data). In total, 108 articles provided records for the Strait of Magellan.

Dubious records were counted as were species that were recorded only once in history. Criteria were followed to determine doubtful species records, as follows: species that were cited once and later questioned in taxonomic revisions or never reported again; species that greatly exceed their distribution limit and do not appear in taxonomic revisions or alpha diversity studies; and species that have a huge geographical discontinuity and are not explained or figured in the article.

A new matrix was elaborated with the Darwin Core standard from the database, with presence-absence data of each taxon per quadrant entered as 1 or 0, respectively. The quadrants with no species were removed from the matrix and species/taxa considered doubtful and/or with imprecise locations were not included in the matrix. However, the above cases were considered in the quantification of total richness. On the other hand, the records up to or above genus level (registered as “indet.” or “sp.”) were not considered as valid species for both species richness values and estimation models, except for those in which the author commented that it could be a new species.

Finally, to detect whether the historial sampling effort was able to estimate all the species of mollusks in the Strait of Magellan, the non-parametric species accumulation models Chao 2 and Jacknife 1 (Burnham and Overton 1978; Burnham and Overton 1979; Chao 1987; Colwell and Coddington 1994) were used to evaluate the sampling effort spatially and estimate the number of species expected theoretically in the Strait of Magellan. These methods require only presence-absence data; Chao 2 is calculated with the species that occur in only one sample (single or singleton species) and those that occur exactly in two samples (doubletons). Jacknife 1 is a more accurate and less biased estimator, since it only uses the number of singletons and the number of samples (Moreno 2001). Complementarily, parametric accumulation models were used to detect whether the historical sampling effort was able to estimate the total species of mollusks (Soberón and Llorente 1993); the linear dependence and Clench models were used. All samples were randomized so as not to affect the shape of the curve (Colwell and Coddington 1994; Moreno and Halffter 2000). The estimation of the coefficients of each nonlinear regression model was done using the Simplex and Quasi-Newton estimation methods of the statistical package STATISTICA 7. For all models, species with imprecise locations were not included.

## Results

**Figure 2.**
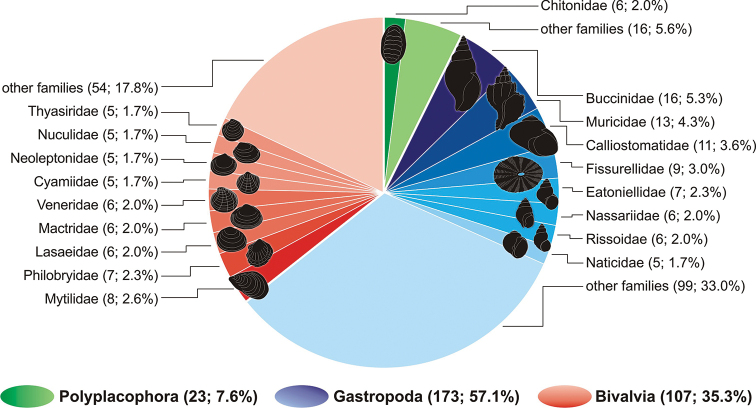
Species richness of mollusks from the Strait of Magellan, highlighting the families with higher diversity. The numbers of species and their percentages are indicated in parentheses.

A total of 134 articles summarizing two centuries of study were entered in the spreadsheet, representing 2579 records corresponding to 412 taxa distributed in the three classes studied (Table 1, Appendix I). Of the total taxa, 65 were reported up to or above genus level (i.e., “indet.” or “sp.”), finding no evidence that they may correspond to new species. On the other hand, 44 species were considered doubtful. Of the total 303 validated species with effective distribution in the Strait of Magellan (Fig. 2), 57.1% belong to the class Gastropoda (173 species); 24.1% of these correspond to the most diverse families: Buccinidae, Muricidae, Calliostomatidae, Fissurellidae, Eatoniellidae, Nassariidae, Rissoidae and Naticidae. The family Buccinidae was the most diverse in the class, with 15 species. The class Bivalvia was represented by 35.3% of the species (107 in total); 17.5% of these correspond to the most diverse families: Mytilidae, Philobryidae, Lasaeidae, Mactridae, Veneridae, Cyamiidae, Neoleptonidae, Nuculidae and Thyasiridae. The remaining 7.6% correspond to the class Polyplacophora (23 species). The family Chitonidae was the most diverse, with 2.0% of the species. In total, 106 families were recorded.

**Table 1.** Species checklist of benthic marine mollusks of the Strait of Magellan (Polyplacophora, Gastropoda and Bivalvia). Those species with a single record are marked with an asterisk (*) and those which are dubious with a square (▪). Their presence is indicated (+) in the eastern (E), central (C) and western (W) microbasins. References provided at the end of the list.

Taxa	Reference	E	C	W
**Class Polyplacophora**				
Polyplacophora indet.	ab, as, bo, cp		+	
Order Lepidopleurida				
Leptochitonidae				
Leptochitonidae indet.	f		+	
*Leptochiton* sp.	cc			
*Leptochiton kerguelensis* Haddon, 1886	t, cd, bm, b, cq, as	+	+	+
*Leptochiton laurae* Schwabe & Sellanes, 2010	cd		+	+
*Leptochiton linseae* Sirenko, 2015	cd		+	
*Leptochiton medinae* (Plate, 1899)	as, cd, cq, bm, h	+	+	+
*Leptochiton smirnovi*▪ Sirenko, 2016	as		+	
*Lepidopleurus cullierti*▪ Rochebrune, 1899	as, bm	+	+	
Order Chitonida				
Ischnochitonidae				
*Ischnochiton* sp.*	e		+	
*Ischnochiton punctulatissimus* (Sowerby I, 1832)	b		+	+
*Ischnochiton pusio* (Sowerby I, 1832)	b, cq, br		+	+
*Ischnochiton stramineus* (G. B. Sowerby I, 1832)	p, cq, cc, b, t, bv, am, ej	+	+	+
*Ischnochiton striolatus*▪ (Gray, 1828)	br			
*Stenosemus exaratus* (Sars G. O., 1878)	cq		+	
Chaetopleuridae				
*Chaetopleura angulata*▪ (Spengler, 1797)	br			
*Chaetopleura isabellei*▪ (d’Orbigny, 1841)	br			
*Chaetopleura peruviana*▪ (Lamarck, 1819)	h, e		+	
Callochitonidae				
*Callochiton bouveti* Thiele, 1906	bm, as	+	+	
*Callochiton gaussi* Thiele, 1908	t, as		+	
*Callochiton puniceus* (Gould, 1846)	am, as, bm, ct, i, b, cq, bn, bo, e, t, br, am, bv, ej	+	+	+
*Callochiton steinenii* (Pfeffer, 1886)	bm, as	+	+	
Chitonidae				
*Acanthopleura granulata*▪ (Gmelin, 1791)	p			
*Chiton* sp.	bl		+	
*Chiton bowenii* King, 1833	b, j, bv, cc, ct, ej	+	+	
*Chiton magellanicus*▪ Gmelin, 1791	dd			
*Chiton magnificus*▪ Deshayes, 1827	h, j		+	
*Chiton olivaceus*▪ Spengler, 1797	p			
*Tonicia* sp.	b, i, j		+	
*Tonicia atrata* (G. B. Sowerby II, 1840)	cq, ct, j, as, bm, bo, e, s, bu, bv, ar, ej	+	+	+
*Tonicia calbucensis* Plate, 1897	cq, j		+	+
*Tonicia chilensis* (Frembly, 1827)	j, as, bm, bu, bv	+	+	
*Tonicia disjuncta*▪ (Frembly, 1827)	as		+	
*Tonicia lebruni* Rochebrune, 1884	bm, cq, ej	+	+	+
*Tonicia smithi* Leloup, 1980	b, cc, bu, bv, b, a, am, cc			+
Mopaliidae				
*Nuttallochiton hyadesi*▪ (Rochebrune, 1884)	p		+	
*Nuttallochiton martiali* (Rochebrune, 1884)	b, cq, br, t, bv		+	+
*Plaxiphora aurata* (Spalowsky, 1795)	bu, bv, cq, bm, bo, e, j, am, a, br, ar, bk, ba, bl, t, i, b	+	+	+
Acanthochitonidae				
Notoplax magellanica* Thiele, 1909	am			
Hemiarthridae				
*Hemiarthrum setulosum* Carpenter in Dall, 1876	br, cc			
**Class Gastropoda**				
Gastropoda indet.	as, j, bo		+	
Order Patellida				
Lottiidae				
*Lottia* sp.	bl, bk, cb		+	
*Lottia orbignyi*▪ (Dall, 1909)	h			
*Scurria ceciliana* (d’Orbigny, 1841)	br, b, a, cs	+	+	+
*Scurria ceciliana magellanica* (Strebel, 1907)	co, de		+	+
*Scurria plana*▪ (Philippi, 1846)	bg			
*Scurria variabilis*▪ (G. B. Sowerby I, 1839)	e		+	
Lepetidae				
Lepetidae indet.*	as		+	
*Iothia emarginuloides* (Philippi, 1868)	co, bm, b, ce, bv, v, ad	+	+	+
Nacellidae				
Nacellidae indet.*	as		+	
*Nacella* sp.*	as, bv, bl		+	
*Nacella* sp. juvenile	b		+	+
*Nacella deaurata* (Gmelin, 1791)	cv, co, as, ab, aa, b, a, bm, aq, cb, bw, e, y, d, bu, bv, ba, bg, bk, bl, br, cd, j	+	+	+
*Nacella flammea* (Gmelin, 1791)	b, bu, bv, bw, j, e, i, y, ar, bk, bl, aa		+	+
*Nacella magellanica* (Gmelin, 1791)	as, bw, an, ah, ai, y, cs, aq, co, b, a, cv, bl, bk, bg, br, e, bu, aa, h, j, ar, cb, ab, d	+	+	+
*Nacella mytilina* (Helbling, 1779)	co, cv, z, bw, cs, bg, i, as, b, bv, br, x, aa, bk, ar, ba, bo, cg, cp	+	+	+
Order Seguenziida				
~Seguenzioidea				
Lissotesta impervia* (Strebel, 1908)	b			+
Order Lepetellida				
Fissurellidae				
Fissurellidae indet.	as		+	+
Diodora patagonica* (d’Orbigny, 1839)	bg		+	
*Fissurella* sp.	as, b, e, bo, bl, ab, j		+	+
Fissurella nigra* Lesson, 1831	k			
*Fissurella oriens* G. B. Sowerby I, 1834	co, b, bu, bv, i, br, bo, ce, ao		+	+
*Fissurella picta* (Gmelin, 1791)	co, bu, e, ar, bo, bk, bl, bg	+	+	
*Fissurella picta picta* (Gmelin, 1791)	a, b, ao, bv	+	+	
*Fissurella radiosa* Lesson, 1831	b, ao, ar, e, br, bu, cr, ba		+	+
*Fissurellidea patagonica* (Strebel, 1907)	bw, ap		+	
*Lucapinella henseli* (Martens, 1900)	k, av		+	
*Parmaphorella* sp.*	as		+	
*Puncturella* sp.	bm, as	+	+	
*Puncturella conica* (d’Orbigny, 1841)	b, f, k, cy		+	+
*Puncturella noachina*▪ (Linnaeus, 1771)	as, co		+	+
Scissurellidae				
*Scissurella clathrata* Strebel, 1908	cz, b, dj, eb		+	
Scissurella petermannensis* Lamy, 1910	cz			
Anatomidae				
*Anatoma conica* (d’Orbigny, 1841)	cz		+	
*Anatoma euglypta* (Pelseneer, 1903)	df			
Order Trochida				
Trochidae				
*Trochidae* indet.*	as		+	
Diloma nigerrimum* (Gmelin, 1791)	h			
Calliostomatidae				
*Calliostoma* sp.*	b			+
*Calliostoma irisans* Strebel, 1905	cl		+	+
*Calliostoma modestulum* Strebel, 1908	bv, as		+	+
*Calliostoma moebiusi* Strebel, 1905	bm, as, l	+	+	
*Calliostoma nudum* (Philippi, 1845)	as, bm, b, j, bv, cl, l	+	+	+
*Margarella* sp.*	as		+	
*Margarella expansa* (G. B. Sowerby I, 1838)	a, b, bv, ci, bt	+	+	
*Margarella jason*▪ Powell, 1951	av, as		+	
Margarella pruinosa* (Rochebrune & Mabille, 1885)	bq, l		+	
*Margarella violacea* (King, 1832)	as, cl, b, bt, av, i, bd, cg, bm, ar, bv, s, bo, bw, e, j, ak, ba	+	+	+
*Photinastoma taeniatum* (G. B. Sowerby I, 1825)	as, bm, bv, bq, f, av, l	+	+	
*Photinula coerulescens* (King, 1832)	br, av, bm, ar, i, as, bn, bk, ce, bg, s, ak, bp, cl, al	+	+	
Photinula crawshayi* E. A. Smith, 1905	cg		+	
*Photinula roseolineata* (E. A. Smith, 1885)	bm, bw	+		
Colloniidae				
*Homalopoma cunninghami* (E. A. Smith, 1881)	bm, as, b, h, cl	+	+	+
Margaritidae				
*Margarites* sp.*	bm	+		
*Margarites sigaretinus*▪ (Sowerby I, 1838)	ci	+		
Tegulidae				
*Tegula atra* (Lesson, 1830)	b, as, bw, j, o		+	+
*Tegula patagonica* (d’Orbigny, 1835)	bg, l	+		
Turbinidae				
Prisogaster niger* (W. Wood, 1828)	h			
Caenogastropoda unassigned				
Turritellidae				
Turritellidae indet.*	as	+		
Epitoniidae				
Epitoniidae indet.	as		+	+
*Cirsotrema magellanicum* (Philippi, 1845)	br, bh		+	
*Cirsotrema strebeli* Zelaya & Güller, 2018	cm, ed		+	+
Newtoniellidae				
*Eumetula michaelseni* (Strebel, 1906)	as, cm, ef		+	
*Eumetula pulla* (Philippi, 1845)	b, bm, bv, as, sm, ce, bh	+	+	+
Order Littorinimorpha				
Eatoniellidae				
*Eatoniella* sp.	as, b, bm	+	+	+
*Eatoniella afronigra* Ponder & Worsfold, 1994	bv, bc		+	
Eatoniella argentinensis* Castellanos & Fernández, 1972	bm	+		
*Eatoniella denticula* Ponder & Worsfold, 1994	bc, b		+	+
*Eatoniella ebenina* Ponder & Worsfold, 1994	bc, b		+	+
Eatoniella glomerosa* Ponder & Worsfold, 1994	bc		+	
Eatoniella picea* Ponder & Worsfold, 1994	bc		+	
*Eatoniella turricula* Ponder & Worsfold, 1994	bc			+
Capulidae				
Capulus compressus* Pelseneer, 1903	m			
*Capulus subcompressus*▪ Pelseneer, 1903	as		+	
Capulus ungaricoides* (d’Orbigny, 1841)	av		+	
Littorinidae				
*Laevilitorina caliginosa* (Gould, 1849)	b, ar, co, bk		+	+
Naticidae				
Naticidae indet.*	as			+
Euspira constricta* Dall, 1908	bh			+
*Falsilunatia carcellesi* Dell, 1990	as, bm, al, dj	+	+	
*Falsilunatia falklandica*▪ (Preston, 1913)	bm	+		
*Falsilunatia patagonica* (Philippi, 1845)	br, av, bw, bh, cn, b, v, i, f, dj, dz		+	+
*Natica* sp.*	s	+		
Natica limbata* d’Orbigny, 1837	cg, dz		+	
*Notocochlis isabelleana*▪ (d’Orbigny, 1840)	bm	+		
*Polinices* sp.	dz			
*Tectonatica impervia* (Philippi, 1845)	bh, cn, bm, v, b, o, dz	+	+	+
Rissoidae				
*Onoba georgiana* (Pfeffer, 1886)	bc		+	
*Onoba lacuniformis* Ponder & Worsfold, 1994	bc		+	
*Onoba schythei* (Philippi, 1868)	b, bc, as, af		+	+
*Onoba subincisa* Ponder & Worsfold, 1994	bc		+	
Onoba sulcula* H. Adams & A. Adams, 1852	b			+
*Powellisetia microlirata* Ponder & Worsfold, 1994	bc, b		+	+
Caecidae				
Caecum chilense* Stuardo, 1962	b			+
*Caecum magellanicum* (di Geronimo, Privitera & Valdovinos, 1995)	dg			+
Cochliopidae				
Littoridina angustiarum* Preston, 1915	bh		+	
Littoridina faminensis* Preston, 1915	bh		+	
Littoridina limosa* Preston, 1915	bh		+	
Littoridina lioneli* Preston, 1915	bh		+	
Hydrobiidae				
*Hydrobia antarctica* Philippi, 1868	bh			
Eulimidae				
*Eulimidae* indet.	as			+
Calyptraeidae				
Calyptraeidae indet.*	as		+	
*Crepipatella* sp.	dh		+	
*Crepipatella dilatata* (Lamarck, 1822)	b, br, ar, e, bw, as, bn, bo, cn	+	+	+
*Crucibulum quiriquinae* (Lesson, 1830)	di			
*Trochita pileolus* (d’Orbigny, 1841)	as, av, bm, bn, b, f, dj, ec	+	+	+
*Trochita pileus* (Lamarck, 1822)	bm, bw, cn, av, as, a, bu, bv, ar, o, i, bn, bh, ce, dj, ec	+	+	+
Velutinidae				
*Lamellaria* sp.*	j		+	
*Lamellaria ampla* Strebel, 1906	dj		+	
*Lamellaria elata* Strebel, 1906	dj, m		+	
Lamellaria hyadesi* Mabille & Rochebrune, 1889	br		+	
*Lamellaria mopsicolor*▪ Ev. Marcus, 1958	dk			
*Lamellaria patagonica* Mabille & Rochebrune, 1889	as, cn		+	+
*Lamellaria perspicua* (Linnaeus, 1758)	dl		+	
*Marseniopsis pacifica*▪ Bergh, 1886	m			
Cymatiidae				
*Argobuccinum pustulosum* (Lightfoot, 1786)	b, s, j		+	+
*Fusitriton magellanicus* (Röding, 1798)	j, b, s		+	+
Order Neogastropoda				
Volutidae				
Volutidae indet.*	as		+	+
*Adelomelon ancilla* (Donovan, 1824)	cn, bi, as, bm, s, av, e, b, br, f, i, ba	+	+	+
*Adelomelon beckii* (Powell, 1951)	bi, cn	+		
*Adelomelon ferussacii* (Donovan, 1824)	s, cn	+	+	
*Odontocymbiola magellanica* (Gmelin, 1791)	as, e, bi		+	
Cancellariidae				
*Admete* sp.*	f		+	
*Admete magellanica* (Strebel, 1905)	as, bm, cm	+	+	+
Admete philippi* Ihering, 1907	s	+		
*Admete schythei* (Philippi, 1855)	b, bi		+	+
Buccinidae				
Buccinidae indet.	as, dj		+	+
*Anomacme smithi* Strebel, 1905	as, bm	+	+	
*Antistreptus magellanicus* Dall, 1902	bi, as, dj		+	+
*Argeneuthria cerealis* (Rochebrune & Mabille, 1885)	b, bv		+	+
Argeneuthria euthrioides* (Strebel, 1905)	cm		+	
*Argeneuthria paessleri* (Strebel, 1905)	cm, b, bv		+	+
*Argeneuthria philippii* (Strebel, 1905)	az, cm		+	
*Falsimacme kobelti* (Strebel, 1905)	cm, az	+	+	+
*Glypteuthria meridionalis* (E. A. Smith, 1881)	as, az, cm, ce		+	+
*Meteuthria martensi* (Strebel, 1905)	cm, az, b	+	+	+
*Microdeuthria michaelseni* (Strebel, 1905)	as, az, b, bm, cm, bv	+	+	+
*Pareuthria atrata* (E. A. Smith, 1881)	as, b, cm, ak, bm, av, az, o, ce, dj	+	+	+
*Pareuthria fuscata* (Bruguière, 1789)	az, j, bw, ar, cm, bu, bv, as, a, f, i, ab, cb, b, bd, bk, e, bn, ak, o	+	+	+
Savatieria areolata* Strebel, 1905	bm	+		
*Savatieria coppingeri* (E. A. Smith, 1881)	as, cm		+	
*Savatieria frigida* Rochebrune & Mabille, 1885	as, cm, dm		+	+
*Savatieria meridionalis* (E. A. Smith, 1881)	b, cm, bv, ce		+	+
Nassariidae				
Buccinanops cochlidium* (Dillwyn, 1817)	c			
Buccinanops deformis* (King, 1832)	c		+	
*Buccinanops monilifer* (Kiener, 1834)	c	+		
*Buccinanops paytensis* (Kiener, 1834)	c, bw,r	+	+	
Nassarius coppingeri*(E. A. Smith, 1881)	b			+
*Nassarius gayii* (Kiener, 1834)	h, r		+	
*Nassarius taeniolatus*▪ (Philippi, 1845)	r			
Muricidae				
*Acanthina monodon* (Pallas, 1774)	bw, e, ar, bu, bk, bl, cg		+	
*Acanthina unicornis*▪ (Bruguière, 1789)	w		+	
*Concholepas concholepas* (Bruguière, 1789)	dn			
*Coronium acanthodes* (Watson, 1882)	ay			+
Enixotrophon veronicae* Pastorino, 1999	ax			+
*Fuegotrophon pallidus* (Broderip, 1833)	as, ce, bm, ar, bv, ak, a, ck, dj, eg	+	+	+
*Tromina* sp.*	bm	+		
*Tromina dispectata* Dell, 1990	cu, q			
*Trophon* sp.	as, ab		+	
*Trophon geversianus* (Pallas, 1774)	b, e, i, j, s, ar, av, ay, ck, ce, cf, bu, bv, bw, br, bk, bl, bi	+	+	+
Trophon minutus* Melvill & Standen, 1907	as		+	
*Trophon ohlini* Strebel, 1904	as, ck, dj, eg		+	
*Trophon plicatus* (Lightfoot, 1786)	ar, ck, av, ce, b, ay, cu, f		+	+
*Xymenopsis buccineus* (Lamarck, 1816)	cn, ak, av, aw	+	+	
*Xymenopsis muriciformis* (King, 1832)	b, ak, ar, as, av, aw, bi, bk, bl, bo, br, bv, bw, cu, ce, cn, eg, p	+	+	+
*Xymenopsis subnodosus* (Gray, 1839)	aw			
Borsoniidae				
*Typhlodaphne filostriata* (Strebel, 1905)	cm, eh		+	+
*Typhlodaphne payeni* (Rochebrune & Mabille, 1885)	b		+	+
*Typhlodaphne strebeli* Powell, 1951	b		+	+
Cochlespiridae				
*Aforia* sp.	bm	+		
Drilliidae				
*Agladrillia fuegiensis* (Smith, 1888)	bm, as, bi	+	+	
*Leptadrillia elissa*▪ (Dall, 1919)	bm, as	+	+	
Mangeliidae				
Belalora cunninghami* (E. A. Smith, 1881)	b, eh		+	
*Lorabela* sp.	bm	+		
*Mangelia martensi* (Strebel, 1905)	do			
*Mangelia michaelseni* (Strebel, 1905)	bm, cm	+	+	
*Oenopota magellanica* (Martens, 1881)	br, cm, dj		+	+
Pseudomelatomidae				
*Leucosyrinx* sp.*	as		+	
Raphitomidae				
*Pleurotomella ohlini* (Strebel, 1905)	cm, eh	+	+	
*Thesbia michaelseni* (Strebel, 1905)	cm, eh	+	+	
Turridae				
Turridae indet.	as		+	
Infraclass “Lower Heterobranchia”				
Mathildidae				
*Mathilda magellanica* Fischer, 1873	b		+	
*Mathilda malvinarum* (Melvill & Standen, 1907)	df			
Cimidae				
*Atomiscala xenophyes* (Melvill & Standen, 1912)	df			
Infraclass Euthyneura				
Acteonidae				
*Acteon biplicatus* (Strebel, 1908)	bm, bv, bj	+	+	
*Acteon delicatus*▪ Dall, 1889	bj			
Ringiculidae				
Microglyphis curtula* (Dall, 1890)	as			+
Order Pleurobranchida				
Pleurobranchidae				
*Berthella platei* (Bergh, 1898)	bn	+	+	
Order Nudibranchia				
Dorididae				
Doris fontainii* d’Orbigny, 1837	by			
*Doris kerguelenensis* (Bergh, 1884)	bx, by, at	+	+	
*Doris magellanica*▪ Cunningham, 1871	s			+
Discodorididae				
*Diaulula hispida* (d’Orbigny, 1834)	by, bx		+	
Diaulula punctuolata* (d’Orbigny, 1837)	by			
Gargamella immaculata* Bergh, 1894	by			
Geitodoris patagonica* Odhner, 1926	by			
Polyceridae				
*Holoplocamus papposus* Odhner, 1926	bx, by, bj		+	+
Thecacera darwini* Pruvot-Fol, 1950	by			
Chromodorididae				
*Tyrinna delicata* (Abraham, 1877)	dp			
Cadlinidae				
*Cadlina magellanica* Odhner, 1926	by, bz		+	
Onchidorididae				
*Acanthodoris falklandica* Eliot, 1907	by, j		+	
Goniodorididae				
Ancula fuegiensis* Odhner, 1926	by			
Janolidae				
*Janolus* sp.*	j		+	
Tritoniidae				
Tritonia australis* (Bergh, 1898)	h		+	
*Tritonia challengeriana* Bergh, 1884	by, bx, j		+	
Tritonia vorax* (Odhner, 1926)	by			
Coryphellidae				
*Itaxia falklandica* (Eliot, 1907)	by, bx		+	
Cuthonidae				
*Cuthona valentini* (Eliot, 1907)	by, bx		+	
Eubranchidae				
Eubranchus fuegiensis* Odhner, 1926	by			
Aeolidiidae				
*Aeolidia* sp.	as, bk	+	+	
*Aeolidia campbellii* (Cunningham, 1871)	by, ar, h, dq		+	
Facelinidae				
Phidiana patagonica* (d’Orbigny, 1836)	bx		+	
Order Cephalaspidea				
Cylichnidae				
Cylichna gelida* (E. A. Smith, 1907)	as			+
*Toledonia* sp.*	as			+
Toledonia parelata* Dell, 1990	bs		+	
*Toledonia perplexa* Dall, 1902	cm, b, n, bj, dj		+	+
Diaphanidae				
*Diaphana paessleri* (Strebel, 1905)	b, dj		+	+
Superorder Sacoglossa				
Plakobranchidae				
*Elysia hedgpethi* Marcus, 1962	bx		+	
Limapontiidae				
Ercolania evelinae* (Marcus, 1959)	bx		+	
*Limapontia* sp.*	bx		+	
Hermaeidae				
*Aplysiopsis brattstroemi*▪ (Marcus, 1959)	bx		+	
Order Siphonariida				
Siphonariidae				
Siphonaria fuegiensis* Güller, Zelaya & Ituarte, 2016	a, ea	+	+	
*Siphonaria laeviuscula*▪ G. B. Sowerby I, 1835	dr			
*Siphonaria lateralis* Gould, 1846	b, co, ar, bk, ab, ea	+	+	+
*Siphonaria lessonii* Blainville, 1824	b, bw, ab, ar, e, co, a, bu, bk, bl, ba, cb, ea	+	+	+
*Williamia magellanica* Dall, 1927	n			+
Superorder Pylopulmonata				
Pyramidellidae				
*Odostomia* sp.	b		+	
*Turbonilla* sp.*	as			+
Turbonilla sanmatiensis* Castellanos, 1982	bm	+		+
*Turbonilla smithi* (Strebel, 1905)	as, bm	+		+
*Turbonilla strebeli* Corgan, 1969	b		+	+
Order Systellommatophora				
Onchidiidae				
*Onchidella marginata* (Couthouy in Gould, 1852)	b		+	
**Class Bivalvia**				
Bivalvia indet.	as, bm		+	
Order Nuculida				
Nuculidae				
*Ennucula eltanini* Dell, 1990	as, v		+	+
*Ennucula grayi* (d’Orbigny, 1846)	as, cw, cp, bn		+	
*Ennucula puelcha* (d’Orbigny, 1842)	t, cw		+	
*Linucula* sp.*	as		+	
*Linucula pisum* (G. B. Sowerby I, 1833)	cw		+	
*Nucula* sp.	as, cp	+		+
*Nucula falklandica* Preston, 1912	b, cw, dj		+	+
Order Solemyida				
Solemyidae				
*Acharax patagonica* (E. A. Smith, 1885)	as		+	+
*Solemya notialis* Simone, 2009	du			
*Solemya occidentalis* Deshayes, 1857	dt			
Order Nuculanida				
Sareptidae				
*Aequiyoldia* sp.*	i		+	
Nuculanidae				
*Nuculana* sp.*	s			+
Propeleda longicaudata* (Thiele, 1912)	cp		+	
Malletiidae				
Malletia chilensis* Desmoulins, 1832	h			
*Malletia inequalis* Dall, 1908	ds		+	
*Malletia subaequalis* (G. B. Sowerby II, 1870)	as, cw, be, f		+	
Neilonellidae				
*Neilonella sulculata* (Gould, 1852)	b, f, as, br, cw		+	+
Siliculidae				
*Silicula patagonica* (Dall, 1908)	as, v		+	+
Tindariidae				
*Tindaria virens* (Dall, 1890)	as			+
Yoldiidae				
*Yoldia* sp.*	as		+	
*Yoldiella chilenica* (Dall, 1908)	as, cw		+	
*Yoldiella granula* (Dall, 1908)	ds		+	
*Yoldiella indolens* (Dall, 1908)	as, cw		+	+
*Yoldiella valettei*▪ (Lamy, 1906)	cp		+	
Order Mytilida				
Mytilidae				
Mytilidae indet.	as		+	
*Aulacomya atra* (Molina, 1782)	bn, j, ab, bw, cb, bo, as, e, bk, bl, bu, bv, bm, ar, ch, ba, u, t	+	+	+
*Choromytilus chorus* (Molina, 1782)	i, bw		+	
*Crenella* sp.*	as			+
*Crenella decussata*▪ (Montagu, 1808)	as			+
*Crenella magellanica* Linse, 2002	b		+	+
*Modiolus patagonicus* (d’Orbigny, 1842)	dt			
*Mytilus chilensis* Hupé, 1854	a, b, e, f, g, j, t, u, ar, ab, as, bk, bl, bm, bo, bp, br, bu, bv, bw, cb, ch, ei	+	+	+
*Mytilus galloprovincialis* Lamarck, 1819	dv, ei		+	
*Mytilus platensis* d’Orbigny, 1842	as, ba		+	
*Perumytilus purpuratus* (Lamarck, 1819)	g, j, ab, e, bk, bl, b, bu, cb, ch		+	+
Order Arcida				
Arcidae				
*Barbatia platei* (Stempell, 1899)	dt			
Limopsidae				
*Limopsis* sp.	as		+	+
*Limopsis hirtella* Rochebrune & Mabille, 1889	as, v		+	
*Limopsis marionensis* E. A. Smith, 1885	as, v, bn		+	+
*Limopsis perieri* P. Fischer in de Folin & Périer, 1870	dt			
Philobryidae				
*Lissarca miliaris* (Philippi, 1845)	b, as, v		+	+
*Philobrya* sp.	bm, b, bv	+	+	+
*Philobrya aequivalvis*▪ (Odhner, 1922)	bm, as	+	+	
*Philobrya antarctica* (Philippi, 1868)	dt			
Philobrya atlantica* Dall, 1896	as			+
*Philobrya blakeana*▪ (Melvill & Standen, 1914)	b, bm	+	+	
Philobrya capillata* Dell, 1964	as		+	
*Philobrya crispa* Linse, 2002	as, bm	+	+	
*Philobrya magellanica* (Stempell, 1899)	as		+	
*Philobrya sublaevis* Pelseneer, 1903	as, bm, be	+	+	
Order Pectinida				
Pectinidae				
Pectinidae indet.	as		+	
*Aequipecten tehuelchus* (d’Orbigny, 1842)	dt			
*Austrochlamys natans* (Philippi, 1845)	b, h, as, bv		+	+
*Chlamys* sp.*	as		+	
*Delectopecten vitreus* (Gmelin, 1791)	as, v			+
*Zygochlamys patagonica* (King & Broderip)	bn, bo, as, h, bm, b, f, bv, be, cx, t, i	+	+	+
Propeamussiidae				
*Cyclopecten* sp.*	as			+
*Cyclopecten subhyalinus* (Smith, 1885)	as			+
Cyclochlamydidae				
*Cyclochlamys multistriata* (Linse, 2002)	b		+	+
Order Limida				
Limidae				
Limidae indet.	as		+	
Acesta patagonica* (Dall, 1902)	bn		+	
*Limea pygmaea* (Philippi, 1845)	as, v, t, b, bv, bm, ch	+	+	+
*Limatula deceptionensis*▪ Preston, 1916	as		+	
*Limatula hodgsoni* (E. A. Smith, 1907)	as, v		+	
Order Lucinida				
Lucinidae				
*Epicodakia falklandica* Dell, 1964	as, b		+	+
*Lucinoma lamellata* (E. A. Smith, 1881)	as, aj, cf		+	+
*Loripes pertenuis*▪ E. A. Smith, 1881	ce, br			
Thyasiridae				
*Adontorhina pisum* (Dall, 1908)	ac, be		+	+
*Parathyasira magellanica* (Dall, 1901)	db		+	
*Thyasira debilis* (Thiele, 1912)	db, cp, as		+	
Thyasira fuegiensis* Dall, 1890	db		+	
*Thyasira patagonica* Zelaya, 2010	dc		+	+
Order Carditida				
Carditidae				
*Cyclocardia compressa* (Reeve, 1843)	as, ce, b		+	+
Cyclocardia thouarsii* (d’Orbigny, 1845)	s			+
*Cyclocardia velutina* (E. A. Smith, 1881)	as, bn, f, bf		+	
Condylocardiidae				
*Carditella exulata*▪ E. A. Smith, 1885	bf		+	
*Carditella naviformis* (Reeve, 1843)	ag, as, bv		+	
*Carditella tegulata* (Reeve, 1843)	b		+	+
*Carditopsis flabellum* (Reeve, 1843)	u, b, ag		+	+
*Carditopsis malvinae*▪ (d’Orbigny, 1845)	as		+	+
Astartidae				
*Astarte longirostra* d’Orbigny, 1842	as, bm, ce, bv, b, u, v	+	+	+
Order Cardiida				
Cardiidae				
*Cardium parvulum* Dunker, 1861	ag			
Tellinidae				
Macoploma inornata* (Hanley, 1844)	br			
Superorder Imparidentia				
Cyamiidae				
*Cyamiocardium* sp.*	as			+
*Cyamiocardium dahli* Soot-Ryen, 1957	b		+	+
*Cyamiocardium denticulatum* (E. A. Smith, 1885)	v, bm, as	+	+	
*Cyamiocardium yeskumaala* Urcola & Zelaya, 2018	dy			+
*Cyamium* sp.*	b		+	
Cyamium antarcticum* Philippi, 1845	br	+		
*Kidderia pusilla* (Gould, 1850)	br			
Gaimardiidae				
*Gaimardia trapesina* (Lamarck, 1819)	b, bw, bv, i, br, cg, ak		+	+
Order Galeommatida				
Lasaeidae				
*Altenaeum mabillei* (Dall, 1908)	be, v			+
*Kellia bullata* Philippi, 1845	bm, br, as	+	+	
*Lasaea adansoni*▪ (Gmelin, 1791)	b		+	+
Lasaea miliaris* (Philippi, 1845)	u		+	
Lasaea petitiana* (Récluz, 1843)	h			
*Mysella* sp.	cp, bm, b	+	+	
*Mysella rochebrunei* (Dall, 1908)	ds		+	
*Pseudokellya cardiformis* (E. A. Smith, 1885)	bm, v, as	+	+	
~Galeommatoidea				
Montacutidae indet.*	f		+	
Order Venerida				
Mactridae				
*Darina solenoides* (King, 1832)	ca, s, br, al, cg	+	+	
*Mactra fuegiensis* E. A. Smith, 1905	ca		+	
*Mulinia byronensis* Gray, 1837	ca		+	
*Mulinia edulis* (King, 1832)	w, s, bw, bf, bm, al, br	+	+	
*Mulinia exalbida* (King, 1832)	s, ca			+
Mulinia levicardo* (E. A. Smith, 1881)	br, ca			
Ungulinidae				
Diplodonta patagonica* (d’Orbigny, 1842)	o			
*Diplodonta punctata*▪ (Say, 1822)	dx			
Veneridae				
Veneridae indet.*	as		+	
*Eurhomalea exalbida* (Dillwyn, 1817)	as, b, bf, i, bm, f, bp, cj,	+	+	+
*Leukoma antiqua* (King, 1832)	b, bw, cj, o		+	+
*Petricola dactylus* G. B. Sowerby I, 1823	dw		+	
*Pitar rostratus* (Philippi, 1844)	b, bf		+	
*Proteopitar patagonicus* (d’Orbigny, 1842)	br			
*Tawera elliptica* (Lamarck, 1818)	bw, cp, bl, b, as, ce, cg		+	
*Venus inflata*▪ King & Broderip, 1832	al		+	
Neoleptonidae				
*Neolepton* sp.	b		+	+
Neolepton amatoi* Zelaya & Ituarte, 2004	b		+	
Neolepton cobbi* (Cooper & Preston, 1910)	as		+	+
*Neolepton concentricum* (Preston, 1912)	b, da, bm, as	+		+
*Neolepton hupei* Soot-Ryen, 1957	as			+
*Neolepton yagan* Zelaya & Ituarte, 2004	b, da		+	+
Order Myida				
Myidae				
Sphenia hatcheri* Pilsbry, 1899	bf	+		
Pholadidae				
*Netastoma darwinii* (G. B. Sowerby II, 1849)	dt			
Teredinidae				
*Bankia martensi* (Stempell, 1899)	h, bf		+	
Order Adapedonta				
Hiatellidae				
Hiatellidae indet.*	as		+	
*Hiatella* sp.	bv, as, ce		+	
*Hiatella antarctica* (Philippi, 1845)	b		+	+
*Hiatella arctica* (Linnaeus, 1767)	as, bu, e, i, u, ar, f, bm, bo, ch	+	+	+
Pharidae				
*Ensis macha* (Molina, 1782)	s, as		+	
Superorder Anomalodesmata				
Pandoridae				
*Pandora braziliensis* G. B. Sowerby II, 1874	br, bm, as, f, ae	+	+	
*Pandora cistula*▪ Gould, 1850	as, br		+	
Lyonsiidae				
*Entodesma cuneata* (Gray, 1828)	dt			
*Entodesma elongatulum* Soot-Ryen, 1957	bm, as	+	+	
Entodesma solemyalis*(Lamarck, 1818)	bf			
Laternulidae				
*Laternula elliptica*▪ (King, 1832)	as		+	
Cuspidariidae				
*Cuspidaria* sp.	as		+	
*Cuspidaria patagonica* (E. A. Smith, 1885)	as, bm, cp, bf	+	+	+
Cuspidaria tenella* E. A. Smith, 1907	as		+	
*Luzonia chilensis* (Dall, 1890)	dt			
Poromyidae				
*Dermatomya mactroides*▪ (Dall, 1889)	as			+
Lyonsiellidae				
*Policordia radiata* (Dall, 1889)	as		+	+

References: **a** (Aldea and Rosenfeld 2011); **b** (Aldea et al. 2011); **c** (Allmon 1990); **d** (Andrade and Brey 2014); **e** (Andrade et al. 2016); **f** (Arntz and Gorny 1996); **g** (Astorga et al. 2007); **h** (Brattström and Johanssen 1983); **i** (Cañete et al. 2014); **j** (Cárdenas 2008); **k** (Castellanos and Landoni 1988); **l** (Castellanos and Landoni 1989); **m** (Castellanos and Landoni 1990); **n** (Castellanos et al. 1993); **o** (Castellanos 1970); **p** (Castellanos 1988); **q** (Castellanos 1992a); **r** (Castellanos 1992b); **s** (Cunningham 1871); **t** (Dell 1964); **u** (Dell 1971); **v** (Dell 1990); **w** (d’Orbigny 1835–1846); **x** (González-Wevar et al. 2010); **y** (González-Wevar et al. 2016a); **z** (González-Wevar et al. 2016b); **aa** (González-Wevar et al. 2017a); **ab** (Guarda 2015); **ac** (Güller and Zelaya 2011); **ad** (Güller and Zelaya 2016a); **ae** (Güller and Zelaya 2016b); **af** (Güller and Zelaya 2017); **ag** (Güller and Zelaya 2013); **ah** (Guzmán and Ríos 1987); **ai** (Guzmán 1978); **aj** (Holmes et al. 2005); **ak** (Hombron and Jacquinot 1854); **al** (King and Broderip 1832); **am** (Leloup 1956); **an** (Mancilla 2010); **ao** (McLean 1984a); **ap** (McLean 1984b); **aq** (Menéndez 2013); **ar** (Mutschke et al. 1998); **as** (OBIS 2018); **at** (Odhner 1926); **av** (Osorio 1999); **aw** (Pastorino and Harasewych 2000); **ax** (Pastorino 1999); **ay** (Pastorino 2005a); **az** (Pastorino 2016); **ba** (Pelseneer 1903); **bc** (Ponder and Worsfold 1994); **bd** (Powell 1951); **be** (Ramírez 1993a); **bf** (Ramírez 1993b); **bg** (Ramírez 1996a); **bh** (Ramírez 1996b); **bi** (Ramírez 1997); **bj** (Ramírez 2000); **bk** (Ríos and Gerdes 1997); **bl** (Ríos and Mutschke 1999); **bm** (Ríos et al. 2003); **bn** (Ríos et al. 2005); **bo** (Ríos et al. 2007); **bp** (Ríos et al. 2010); **bq** (Rochebrune and Mabille 1885); **br** (Rochebrune and Mabille 1889); **bs** (Rosenfeld and Aldea 2011); **bt** (Rosenfeld et al. 2011); **bu** (Rosenfeld et al. 2013); **bv** (Rosenfeld et al. 2015); **bw** (Rosenfeld et al. 2016); **bx** (Schrödl 1996); **by** (Schrödl 1999); **bz** (Schrödl 2000); **ca** (Signorelli and Pastorino 2011); **cb** (Silva 2015); **cc** (Sirenko 2006a); **cd** (Sirenko 2015); **ce** (Smith 1881); **cf** (Smith 1885); **cg** (Smith 1905); **ch** (Soot-Ryen 1959); **ci** (Sowerby 1838); **cj** (Sowerby 1847); **ck** (Strebel 1904); **cl** (Strebel 1905a); **cm** (Strebel 1905b); **cn** (Strebel 1906); **co** (Strebel 1907); **cp** (Thatje and Brown 2009); **cq** (Thiele 1908); **cr** (Tryon and Pilsbry 1890); **cs** (Tryon and Pilsbry 1891); **ct** (Tryon and Pilsbry 1892); **cu** (Tryon 1880); **cv** (Valdovinos and Ruth 2005); **cw** (Villarroel and Stuardo 1998); **cx** (Waloszek 1984); **cy** (Watson 1886); **cz** (Zelaya and Geiger 2007); **da** (Zelaya and Ituarte 2004); **db** (Zelaya 2009); **dc** (Zelaya 2010); **dd** (Kaas et al. 2006); **de** (Nakano and Ozawa 2007); **df** (Di Luca and Zelaya 2019); **dg** (di Geronimo et al. 1995); **dh** (Nuñez et al. 2012); **di** (Dall 1909); **dj** (Linse 2002); **dk** (Marcus 1959); **dl** (Bergh 1898); **dm** (Di Luca and Pastorino 2018); **dn** (Osorio 2002); **do** (Tucker 2004); **dp** (Schrödl 2003); **dq** (Kienberger et al. 2016); **dr** (Álamo and Valdivieso 1987); **ds** (Dall 1908); **dt** (Huber 2010); **du** (Huber 2015); **dv** (Araya 2015); **dw** (Coan 1997); **dx** (Dall 1901); **dy** (Urcola and Zelaya 2018); **dz** (Pastorino 2005b); **ea** (Güller et al. 2016); **eb** (Geiger 2012); **ec** (Pastorino and Urteaga 2012); **ed** (Zelaya and Güller 2017); **ef** (Castellanos 1990); **eg** (Castellanos and Landoni 1993a); **eh** (Castellanos and Landoni 1993b); **ei** (Oyarzún et al. 2016); **ej** (Sellanes 2018).

**Figure 3.**
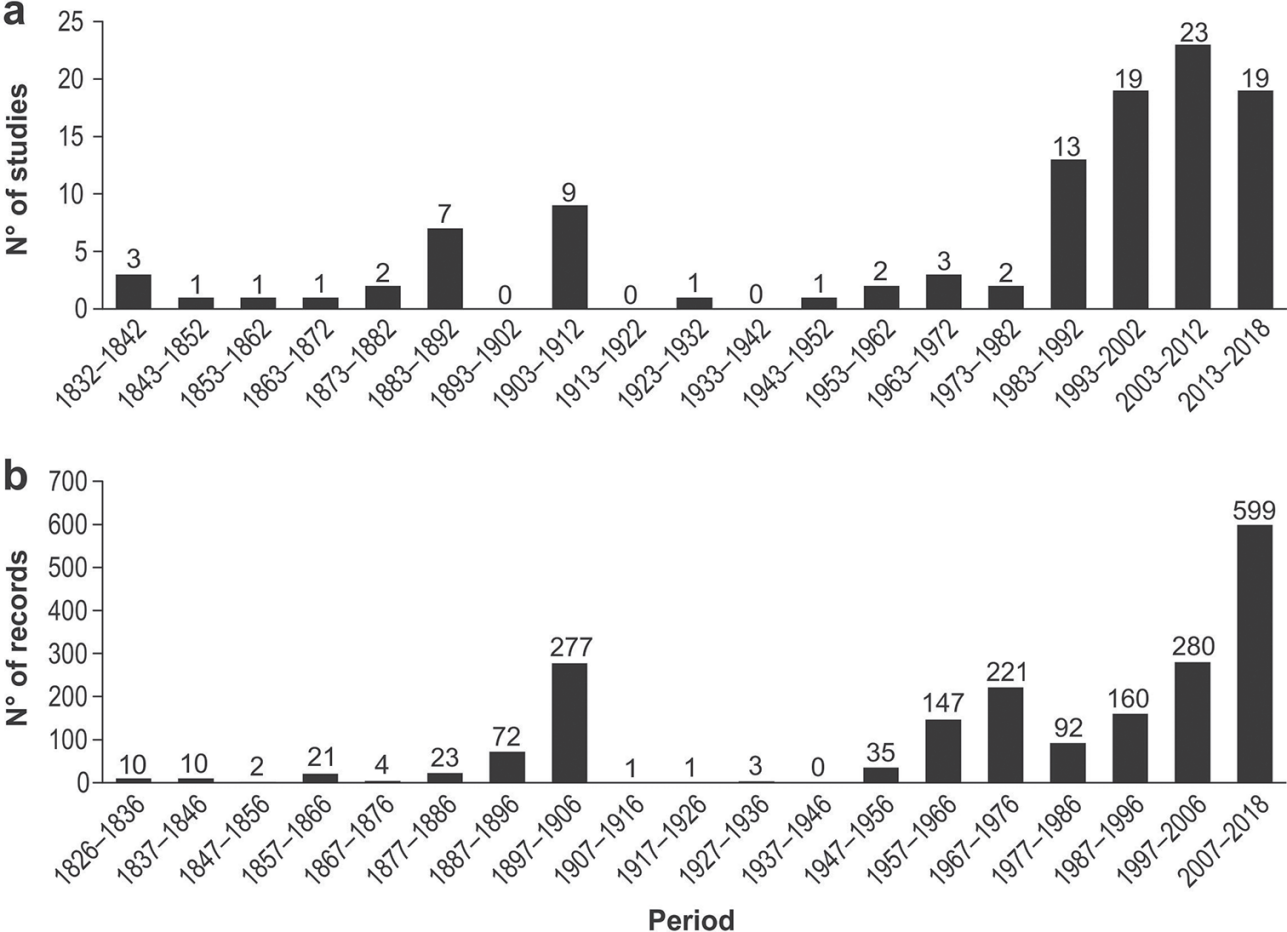
**A** number of studies per decade of the Strait of Magellan mentioned in this study **B** number of mollusk records per decade reported in the Strait of Magellan.

There has been a constant increase since the decade of the 1980s in the number of studies (Fig. 3a) and records (Fig. 3b). The largest number of records in history were incorporated for the Strait of Magellan in the last decade (2007–2018) (Fig. 3b).

**Figure 4.**
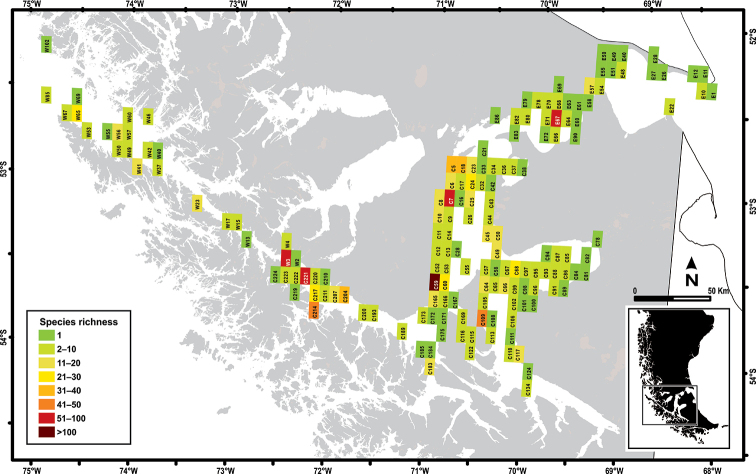
Species richness by quadrant in the Strait of Magellan.

Of the 420 quadrants proposed, 163 presented species (Fig. 4, Appendix II). Ordering the matrix of absence and presence of species according to these quadrants, 1229 mollusk records were counted. The eastern microbasin had 35 quadrants with records, while the central microbasin had 104. The western microbasin proved to be the least historically sampled, with only 24 quadrants with records. The total richness of the Strait of Magellan was 303 species. However, 47 species had imprecise locations, as they were described as inhabitants of the Strait of Magellan, but the site of their habitat was not defined with geographical accuracy. These species include three polyplacophorans (*Leptochiton* sp., *Notoplax magellanica* and *Hemiarthrum setulosum*), 25 gastropods (*Fissurella nigra*, *Anatoma euglypta*, *Scissurella petermannensis*, *Diloma nigerrimum*, *Prisogaster niger*, *Capulus compressus*, *Hydrobia antarctica*, *Crucibulum quiriquinae*, *Buccinanops cochlidium*, *Savatieria frigida*, *Concholepas concholepas*, *Tromina dispectata*, *Xymenopsis subnodosus*, *Mangelia martensi*, *Mathilda malvinarum*, *Atomiscala xenophyes*, *Doris fontainii*, *Gargamella immaculata*, *Diaulula punctuolata*, *Geitodoris patagonica*, *Thecacera darwini*, *Tyrinna delicata*, *Ancula fuegiensis*, *Tritonia vorax* and *Eubranchus fuegiensis*) and 19 Bivalvia (*Solemya notialis*, *Solemya occidentalis*, *Malletia chilensis*, *Modiolus patagonicus*, *Mytilus galloprovincialis*, *Barbatia platei*, *Limopsis perieri*, *Philobrya antarctica*, *Aequipecten tehuelchus*, *Cardium parvulum*, *Macoploma inornata*, *Lasaea petitiana*, *Mulinia levicardo*, *Diplodonta patagonica*, *Proteopitar patagonicus*, *Netastoma darwinii*, *Entodesma cuneata*, *Entodesma solemyalis* and *Luzonia chilensis*).

The quadrants that had species records cover ~37% of the total area of the Strait of Magellan; most of the studies are concentrated in the central microbasin. The quadrant with the highest richness was Punta Santa Ana and Fuerte Bulnes (C59), 60 km south of Punta Arenas with 112 nominal taxa, greatly exceeding the diversity of other quadrants (Fig. 4). The most common species was the gastropod *Nacella magellanica*, present in 33 quadrants, followed by *Pareuthria fuscata* (25 quadrants), *Callochiton puniceus* (23), *Nacella deaurata* (23), *Margarella violacea* (23), *Nacella mytilina* (22), *Trophon geversianus* (22), *Aulacomya atra* (22), *Trochita pileus* (21), *Plaxiphora aurata* (20), *Zygochlamys patagonica* (20), *Mytilus chilensis* (19), *Pareuthria atrata* (18), *Leptochiton kerguelensis* (17), and *Xymenopsis muriciformis* (17).

**Figure 5.**
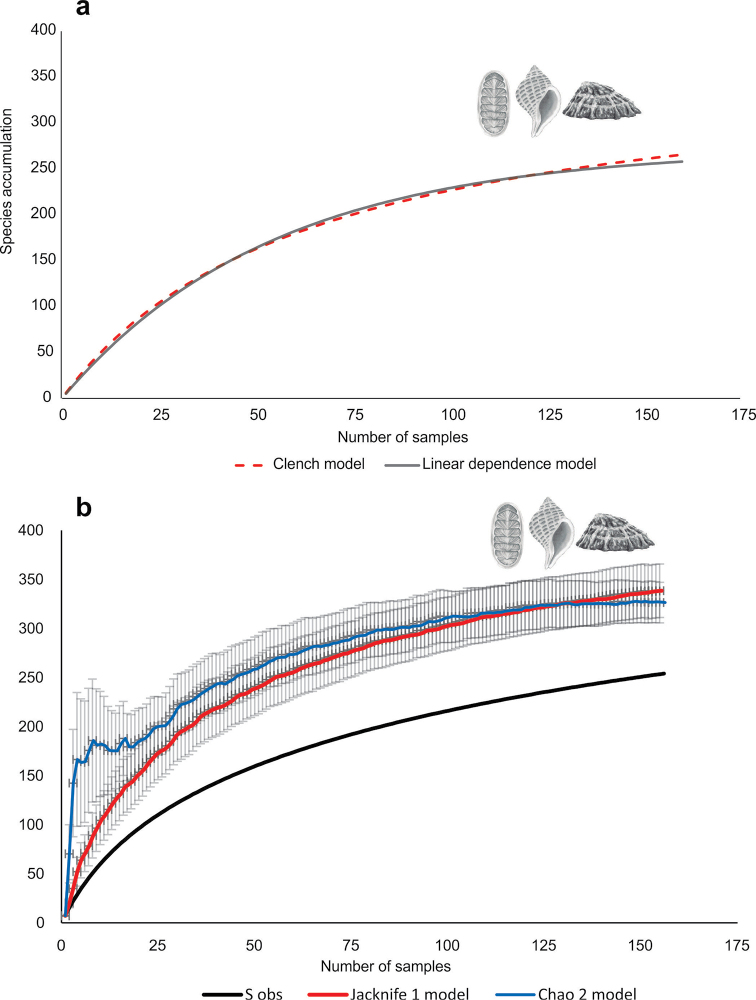
**a** accumulation curves of mollusk species according to the parametric estimators Clench and linear dependence, and **b** according to the non-parametric estimators Chao 2 and Jack 1 for the Strait of Magellan.

The estimated prediction for the richness of species associated with the sampling effort for the Strait of Magellan determined by the Clench model showed that the values of the constants were a = 5.664075 and b = 0.014764. The relation of these values (a / b) obtained a maximum expected richness of 383.6 species (value of the asymptote of the species accumulation curve with R^2^ = 0.97), higher than the 270 species observed. The constants of the linear dependence model were a = 4.953160 and b = 0.017756, thus the maximum expected richness (a / b) was 279 species with R^2^ = 0.97, obtaining a higher value in 9 species than observed in this study (Fig. 5a). Therefore, neither of the two theoretical models predicted exactly the observed number of mollusk species for the Strait of Magellan. Both non-parametric models estimated an expected richness much higher than that observed empirically (Chao 2 = 353.49; Jacknife 1 = 360.39), and both curves were above that of observed richness (Fig. 5b).

## Discussion

**Figure 6.**
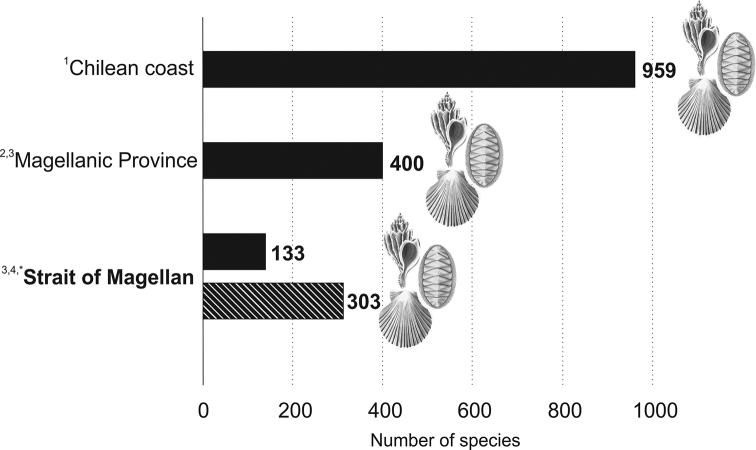
Number of mollusk species cited for the Chilean coast (1: Valdovinos 1999), Magellan Province (2: Linse 1999; 3: Sirenko 2006a) and Strait of Magellan (4: Linse et al. 2006; * this study).

According to Valdovinos (1999), the Chilean coast has about 959 species of the three most diverse classes of benthic marine mollusks (671 gastropods, 226 bivalves and 62 polyplacophorans), including Antarctic and oceanic island species. The Magellan Biogeographic Province (41°S to 56°S) is one of the geographical areas with the highest diversity of mollusks on the Chilean coast (Valdovinos et al. 2003). Taking into account this database, the 303 mollusk species recorded in this study correspond to ~31.6% of the species cited for the Chilean coast (Fig. 6). About 400 species of marine mollusks, 250 gastropods, 131 bivalves (Linse 1999) and 19 polyplacophorans (Sirenko 2006a) have been reported for the Magellan Province. Therefore, the 303 species recorded for the Strait of Magellan represent 75% of the mollusks reported for the MBP. However, comparing the value of richness found in this study (303 species) to the 116 species of gastropods and bivalves reported for the Strait of Magellan by Linse et al. (2006), plus 17 species of polyplacophorans by Sirenko (2006a), the richness of mollusks for the Strait of Magellan was increased by 228% (Fig. 6). Most of the records were reported in the last 70 years. However, records of the late 19^th^ century and early 20^th^ century greatly increased the knowledge of the zone, surpassing previous reports (see Fig. 3). This is mainly due to the publications of Rochebrune and Mabille (1889) and Strebel (1904, 1905a, b, 1906, 1907) which reported 267 records in the Strait. The number of studies has increased in the last 40 years, and therefore the records (see Fig. 3). However, some of these records belong to reviews of biological collections and older studies.

One criterion was followed to determine doubtful species; those records that were cited in the past and have been questioned in taxonomic reviews. Species such as *Carditella exulata* or *Pandora cistula* were identified as dubious according to these criteria (Güller and Zelaya 2013; Güller and Zelaya 2016b). Other criteria included records in which the same taxonomist discussed the species described such as the case of *Doris magellanica* (Cunningham, 1871), records that considerably exceed their distribution limit and do not appear in taxonomic revisions or alpha diversity studies or are simply dismissed, such as *Lottia orbignyi*, *Leptochiton smirnovi*, *Falsilunatia falklandica*, etc. (Espoz et al. 2004; Pastorino 2005b; Sirenko 2016), and records that have a huge biogeographical discontinuity and are not explained or figured in the article, is the case of *Ischnochiton striolatus*, *Puncturella noachina* and *Acteon delicatus* (Rochebrune and Mabille 1889; Strebel 1907; Ramírez 2000). On the other hand, of the taxa reported up to or above genus level (“indet.” or “sp.”), only two could correspond to new species, according to the authors’ remarks: *Leptochiton* sp. (Sirenko 2006a) and *Crepipatella* sp. (Nuñez et al. 2012).

Recent studies using molecular tools have observed that several species co-distributed in the Antarctic Peninsula and South America actually belong to different lineages, with evolutionary units separated by millions of years (Poulin et al. 2014). This has been mainly observed in species of the genus *Aequiyoldia* Soot-Ryen, 1951 (González-Wevar et al. 2019).

Finally, there are species in the list that do not qualify as doubtful, but which have been classified as unknown species due to their low number of records or due to its small body size, which makes it difficult to identify the species, with poor ecological or descriptive information (Castellanos 1979; Geiger 2012; Rosenfeld et al. 2017), e.g., *Notoplax magellanica*, *Lissotesta impervia*, *Onoba sulcula*, *Onoba georgiana*, *Microglyphis curtula*, *Cylichna gelida*, *Turbonilla sanmatiensis*, *Philobrya atlantica*. In this sense, it should be noted that much of the mollusk information that was collected in this work comes from manual collections and various types of sampling gears, trawl and grabs (e.g., Watson 1886; Rochebrune and Mabille 1889; Strebel 1907; Linse 2002; Ríos et al. 2003). However, taxonomic works on specific groups have allowed a good representation of unknown micromollusks (Ponder and Worsfold 1994; Geiger 2012; Pastorino 2016; Di Luca and Pastorino 2018). Despite the aforementioned contributions, micromollusks could continue to be underestimated, since the comparative morphology of various species is only beginning to be illustrated and described in detail (Di Luca and Pastorino 2018).

This historical compilation of the richness of benthic mollusks of the Strait of Magellan promotes the need and urgency for the management of coastal environments. Despite the historical sampling effort and about 192 years of records, the Strait of Magellan has a high diversity of mollusk species which is not yet fully known. The richness estimated by the parametric models was greater than that observed. Two reasons may explain this: i) the sampling effort along the Strait of Magellan has been low (only about 36% of the total area is recorded), and ii) there is still a lack of knowledge about the taxonomy of many mollusk groups, since many species remain undetermined and are not included in the listings or are not recognized in the field. According to Soberón and Llorente (1993), the probability of finding a new species in the Clench model will increase according to experience in the field. Therefore, the Clench model suggests increasing the sampling effort but at a broader spatial and temporal scale to reach the asymptote in the estimation of mollusk species from the Strait of Magellan.

The richness estimated by non-parametric models was higher than the observed. These non-parametric models work based on the number of unique (number of species that occur only in one sample) and duplicate (number of species that occur in exactly two samples). This is based on the assumption that individuals of a species do not live alone in ecosystems, but in populations (Magurran 1988), therefore many unique species in a sample may be indicating that a sufficient number of sampling units has not been used. This historical compilation showed that there are many places in the Strait of Magellan that only have one or two records, which was reflected in both estimators.

However, it is important to consider that in order to evaluate the behavior of the different estimators, it is necessary to know the number of species in the community (Walther and Moore 2005; González-Oreja et al. 2010). Unless the community has been thoroughly sampled, these curves may not work properly (Magurran 2004). Therefore, some authors recommend not working with only one estimator, but testing several models to see how they behave with the data (González-Oreja et al. 2010), since these may vary depending on the situation or for a specific group of organisms (Walther and Moore 2005). The results of the four models used in this study allows us to infer that greater sampling effort is needed in the Strait of Magellan, mainly because the largest number of records and species richness are concentrated at the same points within the Strait of Magellan, in the central microbasin.

## Conclusion

This study provides a clearer idea of the diversity of mollusks in the Strait of Magellan, identifying erroneous records and those that need verification, encouraging other researchers to sample less-studied areas of the strait. This will update knowledge of the diversity of mollusks of the Strait of Magellan, contributing to Chile’s biodiversity heritage and future studies of biogeographical models that are currently based on the 116 species of gastropods and bivalves cited by Linse et al. (2006) and the 17 species of polyplacophorans cited by Sirenko (2006a) for the Strait of Magellan. Finally, with this information of all the records, it will be possible to identify the hotspots of diversity for study and gaps in knowledge, among other things.

## Appendix I

Registration in GBIF database.

Publication date: June 9, 2020

Hosted by: Ministerio del Medio Ambiente de Chile

License: CC BY-NC 4.0

Endpoints: http://gbif-chile.mma.gob.cl/ipt/archive.do?r=moluscos-estrecho-magallanes (Darwin Core Archive), http://gbif-chile.mma.gob.cl/ipt/eml.do?r=moluscos-estrecho-magallanes (EML)

Preferred identifier, DOI: https://doi.org/10.15468/znrbm9

Alternative identifiers: http://gbif-chile.mma.gob.cl/ipt/resource?r=moluscos-estrecho-magallanes

## Appendix II

**Table:** Quadrants of the Strait of Magellan in which mollusks are recorded.

Quadrant	Location	Latitude (S) / Longitude (W)
E1	Dungeness Point 1	52°24’12"S, 68°25’40"W
E10	Dungeness Point 2	52°24’1"S, 68°26’35"W
E11	Dungeness Point 3	52°21’58"S, 68°26’50"W
E12	Dungeness Point 4	52°20’59"S, 68°28’23"W
E22	Point Catalina	52°27’55"S, 68°46’17"W
E26	Cape Posession 1	52°19’40"S, 68°51’5"W
E27	Cape Posession 2	52°19’3"S, 68°56’50"W
E28	Cape Posession 3	52°16’20"S, 69°0’33"W
E40	Posession Bay 1	52°14’25"S, 69°12’30"W
E48	Posession Bay 2	52°17’8"S, 69°12’30"W
E49	Posession Bay 3	52°13’30"S, 69°17’12"W
E50	Tandy Point	52°15’20"S, 69°21’58"W
E51	Posession Bay 4	52°17’8"S, 69°17’17"W
E54	Punta Anegada	52°25’59"S, 69°25’26"W
E55	Nunición Bay	52°20’09"S, 69°26’38"W
E57	Punta Delgada	53°27’12"S, 69°32’7"W
E58	First Narrow 1	52°32’25"S, 69°34’10"W
E60	Punta Remo	52°38’20"S, 69°39’27"W
E61	First Narrow 2	52°32’55"S, 69°40’31"W
E63	Punta Barranca 1	52°32’28"S, 69°43’12"W
E64	Punta Barranca 2	52°37’7"S, 69°43’53"W
E66	Punta Piedras 1	52°44’48"S, 69°50’40"W
E67	Punta Piedras 2	52°38’58"S, 69°50’43"W
E68	Santiago Bay 1	52°34’6"S, 69°50’40"W
E69	Santiago Bay 2	52°29’33"S, 69°51’3"W
E70	Santiago Bay 3	52°31’44"S, 69°55’33"W
E71	Triton Bank 1	52°36’52"S, 69°55’39"W
E72	Triton Bank 2	52°41’44"S, 69°56’6"W
E78	Gregorio Bay 1	52°34’34"S, 70°4’47"W
E79	Gregorio Bay 2	52°35’00"S, 70°08’23"W
E80	Gregorio Bay 3	52°38’13"S, 70°7’58"W
E82	Cape Gregorio	52°39’27"S, 70°14’25"W
E83	Second Narrow 1	52°43’5"S, 70°14’48"W
E86	Second Narrow 2	52°41’44"S, 70°26’17"W
E90	Punta Remo	52°42’43"S, 69°40’28"W
C5	Cabo Negro 1	52°56’30"S, 70°47’46"W
C6	Río Seco	53°2’27"S, 70°49’50"W
C7	Punta Arenas 1	53°8’8"S, 70°51’30"W
C8	Punta Arenas 2	53°11’47"S, 70°55’52"W
C9	Leñadura 1	53°15’24"S, 70°51’35"W
C10	Leñadura 2	53°15’46"S, 70°56’32"W
C11	Santa María Point 1	53°21’57"S, 70°57’37"W
C12	Colorado River 1	53°29’10"S, 70°56’49"W
C13	Colorado River 2	53°28’47"S, 70°51’4"W
C14	Santa María Point 2	53°21’53"S, 70°51’51"W
C16	Paso Ancho 1	53°8’53"S, 70°43’11"W
C17	Paso Ancho 2	53°4’5"S, 70°42’43"W
C18	Cabo Negro 2	52°56’29"S, 70°44’50"W
C21	Marta Island	52°52’57"S, 70°34’48"W
C23	Paso Ancho 3	52°58’19"S, 70°39’54"W
C24	Paso Ancho 4	53°2’11"S, 70°40’1"W
C25	Paso Ancho 5	53°7’34"S, 70°41’34"W
C26	Paso Ancho 6	53°13’4"S, 70°42’24"W
C28	Paso Ancho 7	53°23’35"S, 70°48’47"W
C32	Paso Ancho 8	53°2’15"S, 70°32’49"W
C33	Paso Ancho 9	52°56’34"S, 70°32’5"W
C34	Paso Ancho 10	52°56’19"S, 70°27’31"W
C36	Zegers Point	52°56’20"S, 70°18’52"W
C37	Gente Grande Bay 1	52°55’44"S, 70°12’33"W
C38	Gente Grande Bay 2	52°55’40"S, 70°7’41"W
C42	Gente Point	53°3’13"S, 70°25’45"W
C43	Paso Ancho 10	53°9’47"S, 70°26’17"W
C44	Paso Ancho 11	53°16’46"S, 70°28’16"W
C45	Porvenir Bay 1	53°20’57"S, 70°27’33"W
C49	Paso Boquerón	53°25’59"S, 70°19’40"W
C50	Porvenir Bay 2	53°18’29"S, 70°22’45"W
C52	Carrera Bay	53°33’53"S, 70°54’57"W
C53	Paso del Hambre 1	53°32’47"S, 70°49’20"W
C55	Paso del Hambre 2	53°32’30"S, 70°39’57"W
C57	Cape Valentín 1	53°32’12"S, 70°24’51"W
C58	Inútil Bay 1	53°32’8"S, 70°17’0"W
C59	Santa Ana Point	53°37’55"S, 70°54’41"W
C60	Paso del Hambre 3	53°37’51"S, 70°49’53"W
C64	Cape Valentín 2	53°39’16"S, 70°27’59"W
C65	Inútil Bay 2	53°39’4"S, 70°19’33"W
C66	Inútil Bay 3	53°38’40"S, 70°14’8"W
C67	Cape Boquerón	53°32’26"S, 70°13’43"W
C68	Inútil Bay 4	53°31’49"S, 70°9’20"W
C78	Puerto Nuevo	53°22’23"S, 69°22’14"W
C81	Inútil Bay 5	53°31’36"S, 69°23’42"W
C82	Inútil Bay 6	53°26’59"S, 69°23’58"W
C84	Inútil Bay 7	53°31’5"S, 69°30’41"W
C85	Inútil Bay 8	53°25’58"S, 69°35’25"W
C86	Inútil Bay 9	53°29’40"S, 69°35’4"W
C87	Inútil Bay 10	53°26’28"S, 69°44’32"W
C88	Inútil Bay 11	53°32’24"S, 69°44’48"W
C89	Inútil Bay 12	53°37’18"S, 69°39’42"W
C91	Inútil Bay 13	53°39’9"S, 69°45’59"W
C93	Inútil Bay 14	53°33’13"S, 69°52’27"W
C94	Inútil Bay 15	53°27’20"S, 69°52’32"W
C95	Inútil Bay 16	53°33’38"S, 69°59’57"W
C96	Cameron Point 1	53°39’3"S, 69°59’10"W
C97	Inútil Bay 17	53°35’41"S, 70°7’51"W
C98	Inútil Bay 18	53°40’22"S, 70°8’39"W
C99	Inútil Bay 19	53°40’23"S, 70°15’42"W
C100	Cameron Point 2	53°43’38"S, 69°59’20"W
C101	Cape Nose 1	53°44’21"S, 70°5’37"W
C102	Cape Nose 2	53°45’22"S, 70°10’58"W
C104	Whiteside Channel 1	53°45’35"S, 70°22’4"W
C105	Kelp Point	53°47’10"S, 70°25’49"W
C106	Chown Point	53°52’8"S, 70°10’17"W
C107	Whiteside Channel 2	53°52’7"S, 70°14’29"W
C108	Whiteside Channel 3	53°52’12"S, 70°18’59"W
C109	Harris Bay	53°51’18"S, 70°25’33"W
C111	Cóndor River	53°56’44"S, 70°7’46"W
C113	No Entres Bay	53°58’37"S, 70°21’2"W
C115	Owen Sound 1	53°59’8"S, 70°35’16"W
C116	Owen Sound 2	53°59’14"S, 70°38’46"W
C117	Karukinka Point	54°3’57"S, 70°5’17"W
C118	Whiteside Channel 4	54°4’10"S, 70°8’44"W
C122	Owen Sound 3	54°4’8"S, 70°32’47"W
C124	Port Castillo	54°9’47"S, 69°54’58"W
C134	Alta Island	54°16’21"S, 69°55’49"W
C165	Árbol Point	53°45’50"S, 70°57’51"W
C166	Paso del Hambre 4	53°45’57"S, 70°51’16"W
C167	Lomas Bay 1	53°45’50"S, 70°44’45"W
C169	Lomas Bay 2	53°50’6"S, 70°39’51"W
C171	Amigo Bay	53°51’3"S, 70°52’12"W
C172	Paso del Hambre 5	53°52’12"S, 70°57’27"W
C173	Glascott Point	53°51’45"S, 71°5’25"W
C175	Valdés Point	53°55’9"S, 70°52’54"W
C183	Magdalena Channel	53°55’36"S, 70°56’51"W
C184	Magdalena Sound 1	54°5’5"S, 70°57’30"W
C185	Magdalena Sound 2	54°3’8"S, 71°4’51"W
C189	Paso Froward 1	53°58’35"S, 71°13’35"W
C193	Paso Froward 2	53°51’23"S, 71°31’58"W
C200	Cape Holland	53°50’34"S, 71°37’16"W
C204	Andrés Bay	53°45’50"S, 71°49’0"W
C207	West Point	53°44’38"S, 71°55’28"W
C210	Fortescue Bay	53°42’25"S, 72°1’36"W
C211	Charles Island 1	53°44’22"S, 72°4’14"W
C214	Bárbara Bay	53°48’42"S, 72°9’6"W
C217	Charles Island 2	53°45’25"S, 72°8’42"W
C219	Choiseul Bay	53°45’14"S, 72°19’21"W
C220	Charles Island 3	53°40’56"S, 72°8’34"W
C221	Rupert Island	53°39’55"S, 72°14’14"W
C222	Ballena Sound 1	53°40’38"S, 72°19’31"W
C223	Ballena Sound 2	53°40’9"S, 72°25’25"W
C226	Cape Froward	53°53’52"S, 71°15’9"W
W2	Carlos III Island	53°34’32"S, 72°20’6"W
W3	Paso Tortuoso	53°33’25"S, 72°26’20"W
W4	Jerónimo Channel	53°30’13"S, 72°25’4"W
W13	Spider Island	53°31’14"S, 72°40’26"W
W15	Glacier Bay	53°22’9"S, 72°55’35"W
W17	Paso Largo	53°20’52"S, 73°2’12"W
W23	Lewis Bay	53°15’0"S, 73°19’51"W
W37	Chapman Isles	53°3’18"S, 73°45’13"W
W40	Cape Tamar	52°56’38"S, 73°44’54"W
W41	Brazo Damián	53°1’31"S, 73°55’23"W
W42	Tamar Island	52°55’31"S, 73°50’14"W
W46	Sholl Bay	52°43’42"S, 73°50’16"W
W49	Patranca Island	52°56’46"S, 74°1’59"W
W50	Félix Point	52°56’6"S, 74°8’12"W
W53	Tuesday Bay	52°50’43"S, 74°24’40"W
W55	Paso Tamar 1	52°50’27"S, 74°14’40"W
W56	Paso Tamar 2	52°50’43"S, 74°7’21"W
W57	Paso Tamar 3	52°50’24"S, 74°1’10"W
W60	Paso Tamar 4	52°44’34"S, 74°0’41"W
W65	Cape Pilar 1	52°43’29"S, 74°33’11"W
W67	Cape Pilar 2	52°41’56"S, 74°38’45"W
W69	Western entrance	52°37’40"S, 74°33’38"W
W85	Western entrance	52°33’27"S, 74°45’44"W
W102	Victoria Island	52°18’31"S, 74°50’10"W
